# A case of delayed neurological manifestation following carbon monoxide poisoning in Sri Lanka: epidemiology of exposure and literature review

**DOI:** 10.1186/s40360-019-0295-9

**Published:** 2019-04-05

**Authors:** Prabhashini Kumarihamy, Senanayake Abeysinghe Mudiyanselage Kularatne, Manoji Pathirage, Wasala Mudiyanselage Sujeewa Nilanthi Gunaratne, Roshita Waduge

**Affiliations:** 1 Senior Registrar in Medicine, University Medical Unit, Teaching Hospital Peradeniya, Peradeniya, Sri Lanka; 2 Senior Professor of Medicine and Senior Consultant Physician, Department of Medicine, University of Peradeniya, Peradeniya, Sri Lanka; 3 Consultant Physician & Senior Lecturer, Department of Medicine, University of Peradeniya, Peradeniya, Sri Lanka; 4 Associate Professor, Department of Pathology, Faculty of Medicine, University of Peradeniya, Peradeniya, Sri Lanka

**Keywords:** Carbon monoxide poisoning, Delayed neuropsychiatric sequelae, Epidemiological study, Sri Lanka

## Abstract

**Background:**

Carbon monoxide poisoning is a common emergency worldwide, which carries high morbidity and mortality. Some patients who recover from the insult of acute carbon monoxide toxicity may later develop delayed neuropsychiatric sequelae (DNS) after a lucid period in the form of cognitive impairments, a broad spectrum of neurological deficits and affective disorders. Here, we present the first case of DNS following carbon monoxide poisoning in Sri Lanka and epidemiology of the exposure of nine (9) more victims.

**Case presentation:**

A 55-year-old patient and nine other people developed effects of carbon monoxide poisoning in two different occasions after sleeping few hours in the same room in their work place in Sri Lanka. These patients developed spectrum of symptoms with the acute carbon monoxide poisoning. However, one patient developed neurological deterioration pertaining to delayed neuropsychiatric sequelae (DNS) after 1 month of lucid interval. His MRI scan of the brain showed diffuse high signal intensity involving subcortical white matter, globus pallidus on FLAIR and T2W images. These areas showed high signals in DWI images with no significant changes appreciated on ADC map. There was no abnormal contrast enhancement appreciated in the above areas. EEG showed generalized slow waves. He gradually deteriorated over next 2 weeks, exhibited athetoid movements of his feet and hands and went into rigid akinetic mute state. He could not response to any stimulation and even displayed decorticated-like posture and died. Others had normal MRI brain finding at 8 weeks of acute toxicity and all were neurologically normal after 1 year.

**Conclusion:**

Though, it is uncommon in a tropical country like Sri Lanka, clinicians should have high degree of suspicion with the correct circumstances, as it is a challenge for the emergency physicians, even in countries with higher rate of CO poisoning. The exact mechanisms of acute and delayed toxicity, preventive methods and the suggested treatments are yet to be elucidated and this needs further attention and studies.

## Background

Carbon monoxide (CO) is a colorless and odorless gas where poisoning occurs by inhalation, a common reason to visit emergency department (ED) worldwide having high incidence of morbidity and mortality. Symptoms associated with CO poisoning are intriguingly nonspecific and hence it goes unrecognized, unreported and proper diagnosis is a challenge for the physician. Hence, the real incidence is underestimated worldwide. In the United States, it accounts for more than 50,000 emergency department admissions per year [1].

Even though, it is a rare occurrence in a tropical country like Sri Lanka, the clinicians should have high degree of suspicion when a comatose patient is found in a confined area or when several people experience similar nonspecific symptoms after living in a same confined area. Some patients who recover from the insult of acute CO toxicity may later develop delayed neuropsychiatric sequelae (DNS) after a lucid period in the form of cognitive impairments, a broad spectrum of neurological deficits and affective disorders [2, 3]. So far, there is no specific therapy available for these sequelae.

In this report, we present a case of delayed neurological sequelae following CO poisoning and epidemiology of the exposure with analysis of initial symptoms of CO poisoning. In the literature search, apart from few sporadic news items of CO poisoning related deaths in local newspapers, we could not find published reports of CO poisoning, DNS or epidemiological investigation of such events in Sri Lanka. Further, delayed neurological sequelae of CO poisoning is a rare occurrence even in the world literature. Even though, clinical manifestations of CO poisoning including DNS are well known factual knowledge, arriving at the diagnosis is difficult in clinical practice due to lack of obvious history of exposure. In such situation, clinicians have to do a detective’s job that would be a rewarding exercise. This report gives a narrative description of such saga where diagnosis of CO poisoning was made on in-depth analysis of epidemiological evidence and clinical features. The diagnosis of DNS was made possible by MRI findings that nicely fitted with clinico-epidemiology of the case.

## Case presentation

A 55-year-old man, a social drinker and a technical officer by profession presented to the Teaching Hospital, Peradeniya (THP) in the Central Province of Sri Lanka with a history of headache, gradual alteration of level of consciousness (LOC) and behavioral changes of 3 weeks duration. He was apparently well 7 weeks back and experienced headache, faintishness and vomiting at 2 am while doing a night shift in his work place. After the patient has lost consciousness and become unresponsive. The following day morning, other workers who came for the morning shift found him drowsy and less responsive and took him to the nearest hospital after 6 hours of initial symptoms. On admission, his Glasgow Coma Scale (GCS) was 9/15 and there were no any focal neurological signs. He was haemodynamically stable and respiratory examination was normal with normal pulse- oximeter finding. He continued to have vomiting and diarrhoea over the next 48 h. His full blood count, erythrocyte sedimentation rate (ESR), C-reactive protein (CRP), liver and renal profiles were normal. He was treated with 6 L of oxygen via a face mask and was managed as food poisoning. He was gradually improving and was discharged from the hospital on the 4th day of admission and he was totally normal on discharge. After discharge he was asymptomatic and was doing day to day activities normally.

**Fig. 1:**
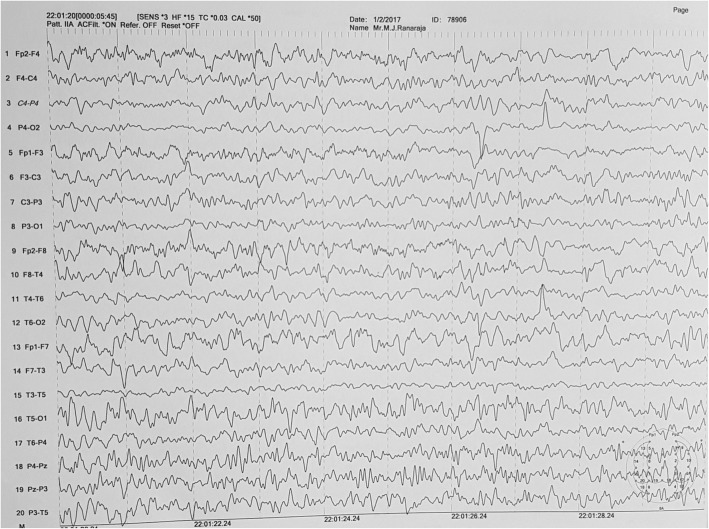
EEG shows grossly irregular back ground rhythm with spikes, sharp waves and slow waves with paroxysmal discharge

**Fig. 2:**
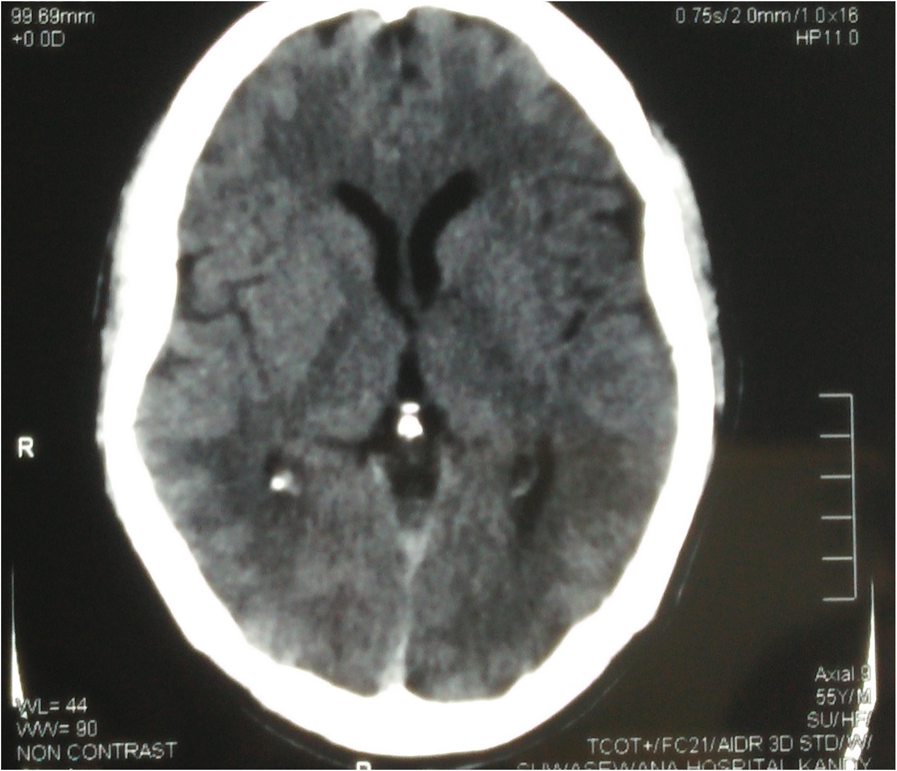
Non contrast computer tomography (CT) of the brain showing prominent cerebral white matter with obliterated sulci

**Fig. 3:**
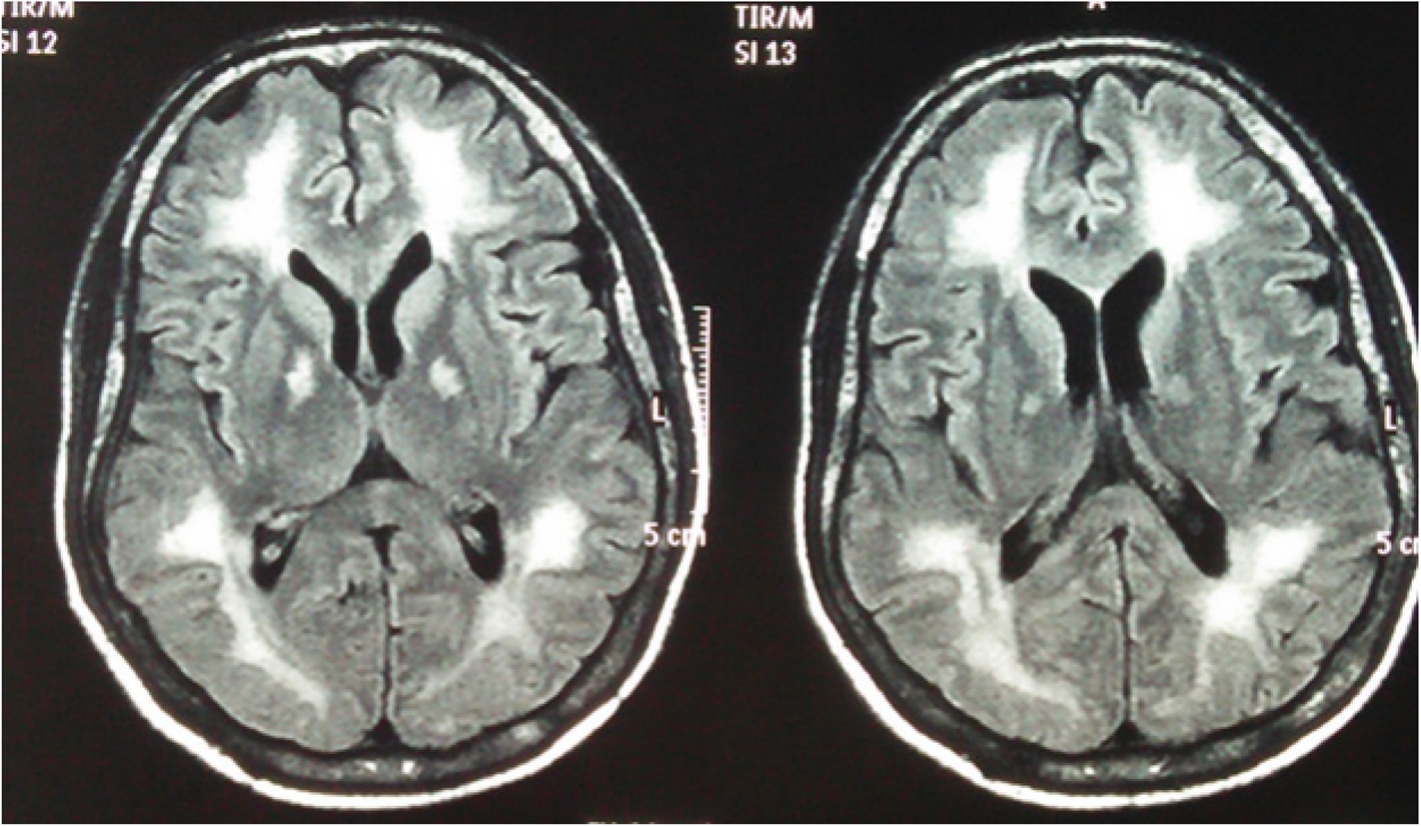
MRI brain shows diffuse high signal intensity involving subcortical white matter, globus pallidus on FLAIR images. Rest of the cerebral hemisphere and ventricular system were normal

**Fig. 4:**
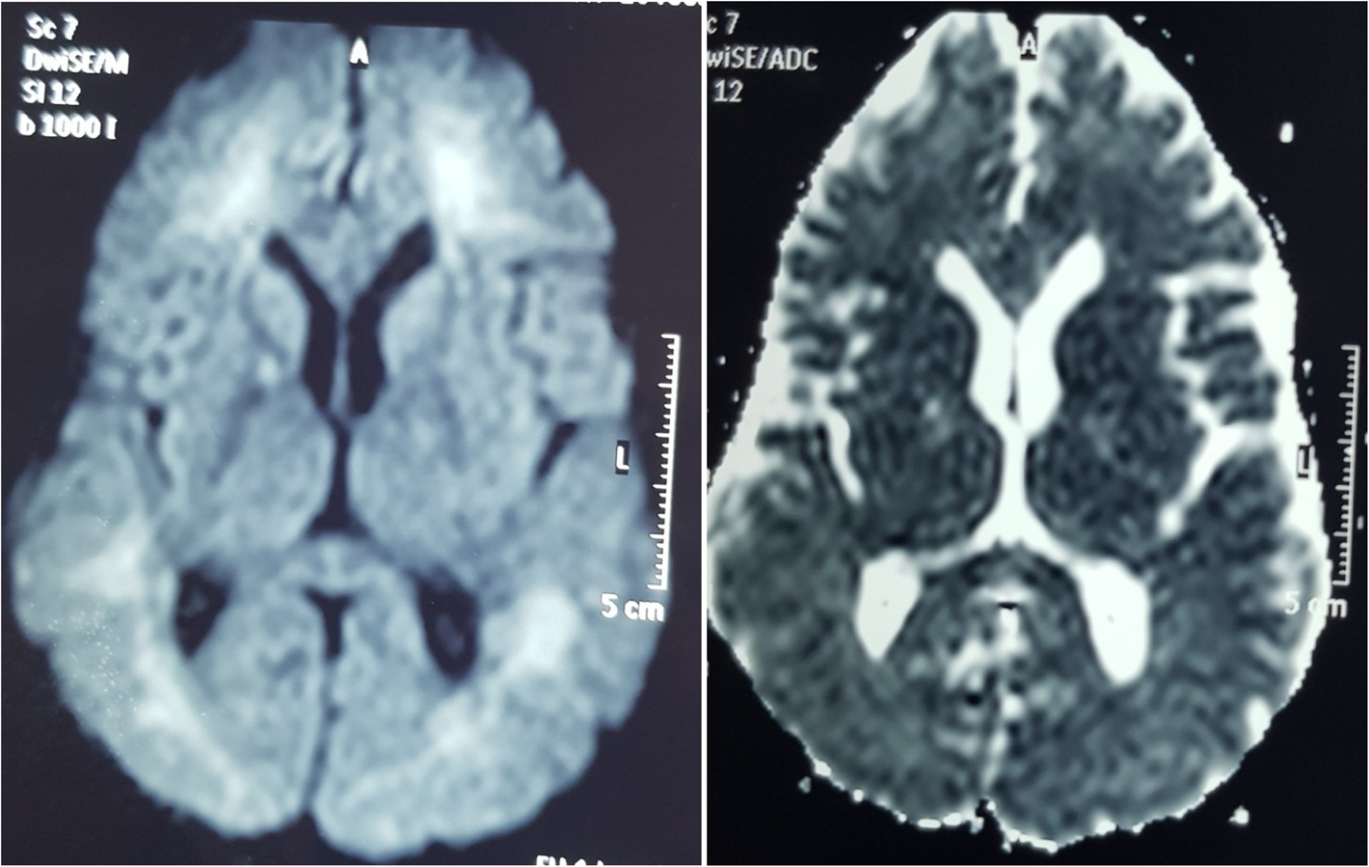
MRI brain showing high signals in DWI images with no significant changes appreciated on ADC map in subcortical white matter, globus pallidus

After 1 month of initial episodes, he developed reduced self-care, a lack of interest of work in which he was interested before the illness. He found it difficult to work as a technical officer due to low energy, lethargy, poor concentration and reduced memory. He was withdrawn at times and was less communicative. He endorsed poor sleep and had a low mood. His condition deteriorated over the next 2 weeks and developed episodic inappropriate talking, confusion, generalized rigidity of the body and urinary and fecal incontinence. He was unable to identify his family members and developed reduced level of consciousness. He was admitted to the THP 7 weeks after the initial incidence. On admission to the THP, he was haemodynamically stable with blood pressure of 140/90. On neurological examination, he was drowsy with GCS of 11/12 (E3, V3, M5). Pupils were normal in size and well reacting to light, both optic fundi were normal, and all cranial nerves were normal. He had generalized rigidity with hyperreflexia and bilateral extensor plantar response. He gradually developed masklike face, positive glabella sign and primitive reflexes (grasp reflex). Mini-Mental State Examination (MMSE) was unable to take because of his demented status. His full blood count, blood picture, ESR, CRP, serum magnesium and calcium levels, liver and renal profiles were normal (Table 1). He had normal electrocardiogram with normal 2D echocardiogram and troponin I was negative. His EEG showed marked generalized slowing of background with multifocal high amplitude waves and delta waves suggestive gross cerebral dysfunction without epileptiform discharges (Fig. 1). Non contrast computer tomography (CT) of brain showed prominent cerebral white matter with obliterated sulci with preserved gray-white demarcation and normal ventricular system with no evidence of intra cerebral haemorrhage (Fig. 2). His cerebrospinal fluid (CSF) report was normal and CSF was negative for both bacterial studies (light microscopy examination and culture) and viral studies (HSV/Entero/Flavi). The MRI brain showed diffuse high signal intensity involving subcortical white matter, globus pallidus on FLAIR and T2W images (Fig. 3). These areas showed high signals in DWI images with no significant changes appreciated on ADC map (Fig. 4). There was no abnormal contrast enhancement appreciated in the above areas. Rest of the cerebral hemisphere, ventricular system, brain stem and cerebellum were normal. While investigating this patient (index case) we came to know that another person (2nd case), who did a night duty with our patient and slept in the same room, also had been admitted with our patient to the same local hospital with the initial incidence. He (2nd case) too has experienced vomiting, diarrhoea, faintishness and unsteady gait, but no changing sensorium. On admission to local hospital his (2nd case) unsteady gait improved, and he had vomiting and few more episodes of diarrhoea. He was totally normal on next day and was discharged from the hospital with a diagnosis of food poisoning. Since then he was asymptomatic.

**Table 1:** Laboratory investigations and Chest radiograph during stay in two hospitals (Local and Teaching Hospital Peradeniya)

	At 1st presentation to local hospital (10th November 2016)	At 2nd presentation to THP (28th December 2016)							Reference value
		Day 1	Day 2	Day 3	Day 5	Day 8	Day 12	Day 14	
White blood cell	10	9.8	–	10.47	11.5	15.8	16.3	14.5	4–10 10^3^ /uL
Haemoglobin	15.7	15	–	15.5	15	14.3	13.2	13.1	11–16 g/dl
Platelets	291	200	–	154	160	200	130	138	150–450 10^3^ /uL
AST	44	–	44	–	–	–	70	45	0–40 umol/l
ALT	35	–	28	–	–	–	51	41	0–40 umol/l
Serum creatinine	99	88	–	102	107	–	108	122	59–104 umol/l
C-reactive protein	5.1	–	5.5	–	9.0	–	20	18	0–10 mg/l
Erythrocyte sedimentation rate mm 1^st^hr	5	–	9	–	6	–	–	–	3–13 mm
Serum sodium	140	144	138	140	142	–	145	148	135–145 mmol/l
Serum potassium	4.6	4.05	4.57	4.7	4.1	–	4.3	4.2	3.6–5 mmol/l
Serum calcium		2.33	2.4	–	2.4	–	–	–	2.2–2.6 mmol/l
Serum magnesium	–	–	0.89	–	1.04	–	–	–	0.73–1.06 mmol/l
CSF analysis	–	Appearance-Colorless transparent; CSF protein 34 (15–45 mg/dl); WBC 1 (100%Lymp (0–5 Cu mm)); CSF glucose-3.2 mmol/l (Random blood sugar 5.1); CSF viral studies (HSV/Entero/flavi), *Mycobacterium tuberculosis* smear (Acid-fast stain), CSF Culture: all negative							
Chest X-ray		–	Normal	–	–	–	Right upper and middle lobe opacities		

*AST* Aspartate aminotransferase, *ALT* Alanine aminotransferase, *CSF* cerebrospinal fluid, *WBC* white blood cells, *HSV* Herpes simplex virus

Further inquiry revealed an another incidence happened in the same place after 2 weeks of initial incidence when 8 workers who came from their main office (Colombo) slept in the same room and all of them became ill after being there for about 3 hours. The most common symptoms were headache and faintishness which were experienced by all 8 workers. Six of them had unsteady gait and four of them fallen on ground when they attempted to walk. Five of them experienced lethargy and nausea and two had vomiting. Loss of consciousness was experienced by three patients and duration of loss of consciousness (LOC) was less than 5 minutes in two of them and around 20 min for the other one. Even though, it was impossible to recall the associated seizure by others, one had urinary and fecal incontinence at the time of LOC. Three people had diarrhoea and two experienced burning sensation of throat. All of them were taken out and two became totally normal within 30 min. Another two became normal within 2 hours. Two patients out of three who developed LOC became asymptomatic after 12 h and they were treated with face mask oxygen and other supportive care at the same local hospital. All the symptoms except headache experienced by the person who had LOC of around 20 min settled after 24 h and his headache lasted for 3 days. The remaining patient had headache for 3 days even though his initial symptoms were mild. All of them were discharged from the local hospital on the same day evening.

After this incidence, the work place was examined by the authorities and found to have newly implanted petrol driven generator, in the ground floor of the two-story building in which all victims (patients) slept. It was found that mal functioning generator emitting carbon monoxide when it was on. In both incidences, there had been a power failure requiring power to be generated by this offending generator. Subsequently, we traced all exposed victims and examined them, including the person who developed symptoms in initial incidence with our patient (index case), after 8 weeks of initial exposure. All of them were neurologically normal and all had normal MRI brain imaging.

Depending on the available history, examination, epidemiology of the incidence and the MRI brain finding, the diagnosis of Carbon Monoxide-induced Delayed Neuropsychiatric Sequelae was made. Supportive management was offered to him. He was started on Sinemet (carbidopa levodopa) up to maximum dosages with no clear therapeutic benefit. Dexamethasone was also tried without obvious effect. He was gradually deteriorated over next 2 weeks, exhibited athetoid movements of his feet, and hands and went to rigid akinetic mute state. He could not respond to any stimulation and even displayed decorticate-like posture. His illness was complicated with aspiration pneumonia and died nearly after 3 weeks of admission. His post mortem examination showed evidence of pneumonia mainly in right lung and punctuate haemorrages in the sub cortical white matter of the brain. Histological examinations of all the organs were normal including brain.

All the others who had exposure history were followed up for 1 year and they were totally asymptomatic at 1 year of initial incidence.

## Discussion and Conclusions

Carbon monoxide poisoning, one of the most common fatal poisonings, occurred with inhalation of a colorless, odorless and non-irritating gas which causes organ damage as a result of cellular oxygen depletion leading to various systemic, neurological complications and death [2]. The Center for Disease Control reports that nearly 500 unintentional deaths occur in the United States each year due to CO poisoning [4]. House fires and improperly vented automobiles, gas heaters, furnaces, hot water heaters, wood- or charcoal-burning stoves, and kerosene heaters are common sources of CO poisoning. In our case CO was emitted by a petrol driven generator mount in a poorly ventilated room in a working place.

It is a well-known fact that CO poisoning is very difficult to diagnose, unless suspected, because the clinical presentation mimics other common illnesses [5, 6] and hence it is commonly missed as a diagnosis [6]. Further, patients can have vague, nonspecific, and variable symptoms making the diagnosis more difficult [6, 7]. The symptoms of CO poisoning may mimic a flu like syndrome or acute gastroenteritis especially in children [8] and however this is true for adults as well. Our indexed case and his work mate also had features of gastroenteritis in the initial presentation and both of them were treated for suspected food poisoning supporting above statement. They had their dinner together which supported the diagnosis of food poisoning at initial presentation. However, the initial loss of consciousness of our indexed patient was not explained with food poisoning. In the second incidence, most of them had symptoms of nonspecific flu or gastroenteritis though a few had neurological symptoms as well which were not explained by a simple flu or gastroenteritis. Physicians should have high degree of suspicion about CO exposure especially if people from the same vicinity experience nonspecific symptoms. Though, they were managed symptomatically, this incidence has led to further investigation of the circumstance and finding out a CO emitting generator situated within the ground floor of two story building. All these people slept in the first floor of this building which was poorly ventilated and there was a power failure on both these occasions requiring power to be generated from this newly installed generator. The generator was removed and there were no further incidents after that. We did not measure CO in the blood in the index case and other exposed victims due to delayed presentation of weeks to THP. Thus, the diagnosis was purely based on clinical and epidemiological evidence as per we have highlighted above. Even though, incidences of CO poisoning and related deaths were reported in newspapers on several occasions [9–11], this is the first such reported incidence in medical literature in Sri Lanka. Further, we could not find any statistical data related to CO poisoning and related deaths in our country. There are many learning points about clinical presentation and epidemiology in this story of CO poisoning in Sri Lanka. Also it unravels the deficiencies in safety of workers and overlooking the diagnosis at the first contact hospital.

The clinical course of the CO poisoning could be either monophasic or biphasic. In the biphasic course, patients can develop delayed neurological sequelae (DNS) after a lucid interval (from days to weeks, usually within 1 month) in which the patient is apparently normal [2, 3, 12]. Hence, DNS is a real concern in people who survived after acute CO poisoning. After acute CO poisoning, most of them recover fully without any further complication and nearly 10 to 30% of victims admitted again to a hospital with DNS [3, 13]. According to a study done in Korea, out of 2759 patients, 75 patients developed DNS [2]. In our case series, all patients had monophasic course except our indexed victim who developed delayed neurological sequelae and this is the first reported case of DNS following CO poisoning in Sri Lanka. In accordance with the medical knowledge, this patient had nearly a 1 month of lucid interval before developing delayed neurological sequelae.

CO has very high affinity to haemoglobin compared to oxygen and it binds to haemoglobin 240 times greater than that of oxygen. With the exposure to CO, there is a competitive binding of CO to haemoglobin, which in turn reduces the oxygen-carrying capacity of the hemoglobin. This causes the shifting of oxygen-hemoglobin dissociation curve to the left and as a consequence, oxygen release at the tissue level is impaired causing hypoxic stress at cellular level [14]. However, exact pathogenic mechanism of acute toxicity of CO and particularly the delayed toxicity and subsequent DNS remain poorly understood even today [16]. The Pathophysiology of DNS is complex and it cannot be fully explained from tissue hypoxia and impaired oxygen transport to the cells [15]. Also CO poisoning causes abnormal inflammatory response through various pathways other than the cellular hypoxia [16]. Hence, more studies are needed to fill the gaps in Pathophysiological mechanism of acute and subsequent effects of CO poisoning.

DNS can manifest with various symptoms including broad spectrum of neurological deficits, such as Parkinsonism, urinary and fecal incontinence, dementia, cognitive impairments and psychosis [3, 16]. Our indexed patient also had features of Parkinsonism, cognitive impairments, urinary and fecal incontinence and some features of psychosis. Depending on the available history, clinical presentation, epidemiology of the incidence and the MRI brain finding, the diagnosis of Carbon Monoxide-induced Delayed Neuropsychiatric Sequelae was made in our patient.

In CO poisoning, the DWMRI shows hyperintense signals in the periventricular and deep white matter [17]. The high signal intensity means cytotoxic oedema may be differentiated from apoptosis and triggered by hypoxia [17]. CO intoxication is thus important in the differential diagnosis of diffused white matter lesions of the cerebrum. Our patient also had similar changes leading to the diagnosis. Hurley et al. mention that the globus pallidus was the most commonly affected area compared to the remaining brain during the first week following acute CO poisoning. This was explained by their high oxygen consumption [18, 19]. This was supported by Parkinson et al. and some other researchers and they mention that acute brain lesions are observed mainly in basal ganglia symmetrically and corpus callosum, and particularly in bilateral globus pallidus. However, during the delayed phase, diffused inflammations in centrum semiovale or periventricular areas of the deep white matter are observed [20, 21].

Even though, some variables have been associated with DNS: initial clinical presentation of the victim poorly correlates with the development of DNS. Some studies have shown several factors predicting the development of DNS; older age, duration of exposure to CO, longer time to treatment, transient loss of consciousness, coma, increased serum levels of neuron specific enolase (NSE), increased levels of myelin basic protein in the cerebrospinal fluid are some of them [3, 22, 23]. Low GCS or severe loss of consciousness (LOC) can be used to predict the development of DNS in respect to several other studies [24, 25]. CO-Hb concentration was not a predictive factor for DNS [25–27]. Hence, LOC can be considered as a predictor for DNS in our indexed patient. However, several other patients also had LOC but they did not develop DNS. Probably this could be explained from the severity and duration of LOC in the indexed case.

Even though, there was no specific treatment for DNS, we treated him with Levodopa, steroid and memantine without any clinically detectable improvement. Levodopa and anticholinergic drugs have been reported ineffective in previous reports as well [28] in reversing the Parkinsonian features. Steroids have been used in a few previous cases in combination with memantine successfully. However, we were not successful with the above regime.

We could not find any clinical features or MRI finding related to the DNS in other nine people and they were healthy at 1 year follow up too.

With the extensive literature survey, we understood that though CO poisoning and related complications are common, the exact mechanisms of acute and delayed toxicity, preventive methods and the suggested treatments are yet to be elucidated [29, 30]. A targeted preventive treatment of DNS during interface between acute poisoning and DNS needs to be worked out. A recent meta-analysis reveals that, neuropsychological sequelae of CO poisoning could be significantly reduced with one session of hyperbaric oxygen (HBO), but the effect of HBO on DNS remains unclear [31]. Therapies targeting inflammatory and oxidative effect of CO and CO scavenging agents are the future directions of therapy [32]. Further, though CO poisoning is uncommon in a tropical country like Sri Lanka, clinicians should have high degree of suspicion with the correct circumstances as it is a challenge for the emergency physicians even in countries with higher rate of CO poisoning. Reporting of such cases are important in further understanding of the CO poisoning associated toxicity and the clinico-epidemiology. And also it may help to increase awareness among general public as well as in medical persons, which will eventually help to reduce the incidence and complications of CO poisoning.

## Abbreviations

CO: Carbon monoxide
CRP: C-reactive protein
CSF: Cerebrospinal fluid full
CT: Computer tomography
DNS: Delayed neuropsychiatric sequelae
ED: Emergency department
EEG: Electroencephalography
ESR: Erythrocyte sedimentation rate
GCS: Glasgow coma scale
HSV: Herpes simplex virus
LOC: Loss of consciousness
MMSE: Mini-Mental State Examination
MRI: Magnetic resonance imaging
NSE: Neuron specific enolase
